# Poor growth response during the first year of growth hormone treatment in short prepubertal children with growth hormone deficiency and born small for gestational age: a comparison of different criteria

**DOI:** 10.1186/s13633-018-0064-3

**Published:** 2018-10-22

**Authors:** Saartje Straetemans, Muriel Thomas, Margarita Craen, Raoul Rooman, Jean De Schepper, BESPEED, A. France, H. Dotremont, M. Den Brinker, M. Cools, K. De Waele, S. Van Aken, S. van der Straaten, I. Gies, J. Vanbesien, J. P. Bourguignon, M. C. Lebrethon, A. S. Parent, C. Heinrichs, S. Tenoutasse, C. Brachet, E. Boros, M. Maes, V. Beauloye, P. Lysy, G. Massa, R. Zeevaert, F. de Zegher, I. Francois, D. Beckers, M. Van Helvoirt, K. Casteels, D. Beckers, T. Mouraux, K. Logghe, G. Thiry-Counson, O. Chivu, S. Depoorter

**Affiliations:** 1 Department of Pediatric Endocrinology, Maastricht University Medical Center, P. Debyelaan 25, 6229 HX Maastricht, The Netherlands; 2 NUTRIM School of Nutrition and Translational Research in Metabolism, Maastricht University, Universiteitssingel 40, 6229 ER Maastricht, The Netherlands; 3 The BElgian Society for PEdiatric Endocrinology and Diabetology (BESPEED), Laarbeeklaan 101, 1090 Brussels, Belgium; 4 Department of Pediatric Endocrinology, University Hospital Ghent, Corneel Heymanslaan 10, 9000 Ghent, Belgium; 5 Department of Pediatric Endocrinology, University Hospital Brussels, Laarbeeklaan 101, 1090 Brussels, Belgium

**Keywords:** Growth hormone treatment, Growth hormone deficiency, Small for gestational age, First-year response, Children

## Abstract

**Background:**

There is no consensus on the definition of poor growth response after the first year of growth hormone (GH) treatment. We determined the proportion of poor responders identified by different criteria in children with GH deficiency (GHD) and born small for gestational age (SGA). The second aim was to analyze the IGF-1 response in poor growth responders.

**Methods:**

First-year height data of 171 SGA and 122 GHD children who remained prepubertal during the first GH treatment year were retrieved from the BESPEED database and analyzed. Criteria for poor first-year response/responsiveness were: change in height (∆Ht) SDS<0.3 or<0.5, height velocity (HV) SDS<0.5 or <1 based on the population reference, HV SDS<− 1 based on the KIGS expected HV curve (HV Ranke SDS), studentized residual (SR) <− 1 in the KIGS first-year prediction model.

**Results:**

∆Ht SDS<0.5 gave the highest percentage poor responders (37% SGA, 26% GHD). Although % poor responders were comparable for ∆Ht SDS<0.3, HV SDS<+ 0.5, HV SDS<+ 1, SR<− 1, and HV Ranke SDS<− 1, these criteria did not always identify the same patients as poor responders. Among the poor growth responders 24% SGA and 14% GHD patients had an IGF-1 increase < 40%.

**Conclusions:**

The different response criteria yield high but comparable percentages poor responders, but identify different patients. This study does not provide evidence that one criterion is better than another. A limited IGF-1 generation is not the major reason for a poor growth response in the first year of GH treatment in SGA and GHD children.

**Trial registration:**

Retrospectively registered.

## Background

Growth hormone (GH) deficiency (GHD) and short stature as a consequence of a small size at birth (SGA) are the most frequent indications for GH therapy in children in Europe. Although in general a substantial fraction of the height deficit is already recovered during the first year of GH treatment in these growth disorders, a high proportion has a poor growth response in the first year of GH therapy [1]. This first year growth response is paramount since it is the major determinant of the gain during the subsequent treatment years and correlates with the final height outcome [2–12].

Traditionally the growth response during the first year of GH treatment is evaluated by auxological parameters, such as the gain in height SDS (∆Ht SDS), the observed height velocity (HV) expressed in cm/year or in SDS, or the increase in HV (∆HV) compared to the pre-treatment year [13]. A number of definitions of poor first-year growth response have been proposed in clinical trials and consensus statements, such as a gain in height <0.3 SD or <0.5 SD, a first-year HV <+ 0.5 SD or <+ 1.0 SD for age and gender, or an increase in HV <3 cm/year compared to the pretreatment year [14].

Another more recent method to evaluate the growth promoting efficacy of GH treatment in short children is to compare the observed to the expected growth response defined by certain patient and treatment characteristics, which has been defined as responsiveness, reflecting the ability of an individual person to respond to GH [11, 12, 15]. First year height velocity response curves, determined by age, treatment indication and sometimes gender (Bakker et al. [16], Ranke et al. [13], and Straetemans et al. [17]) have been published. A height velocity below − 1.0 SD on the growth response curve has been considered as a poor response.

In an attempt to include even more parameters to determine the responsiveness to GH, Ranke et al. have derived prediction models for the first year response to GH in various treatment indications. They include among other factors birth weight, GH dose and parental heights [18–20]. Responsiveness is expressed as a studentized residual [SR = (observed HV – predicted HV)/SD of the predicted HV] and a SR <− 1 has been considered a poor response [12]. This implies that 16.5% of the patients are poor responders. Although these multivariate prediction models provide a more individualized response target, some patients meet their very poor prediction and are therefore not considered poor responders despite their poor absolute response.

Several conditions might explain a poor growth response to GH administration. With the exception of a poorly responsive growth plate, most conditions such as poor compliance, a hidden chronic disease or a partial GH insensitivity due to abnormalities in the GH-IGF-1 axis will limit a sufficient generation of IGF-1 during GH administration. Different patterns of IGF-1 increase during GH treatment between children with GHD, SGA children and other disorders have been described previously [21, 22]. However, up to now, there have been no previous reports comparing the commonly used measures of poor growth response with measures of poor responsiveness from prediction models and only limited data are available on the IGF-1 increase during GH treatment in relation to the growth response in short GHD and SGA children.

We therefore compared the first year growth response and responsiveness criteria in prepubertal children with SGA and GHD, registered in the database of the BElgian Society for PEdiatric Endocrinology and Diabetology (BESPEED). We expected a lower percentage of poor responders using more individualized growth response targets, especially in the SGA group, where GH sensitivity and treatment modalities are more variable. In addition, we evaluated the IGF-1 response during the first year of GH treatment in those children with a poor growth response.

## Methods

### Subjects

The auxological data and first year treatment characteristics of prepubertal children diagnosed with SGA and non-acquired GHD, who had been treated exclusively with recombinant human GH on a daily basis, were retrieved from the Belgian Registry of children treated with GH (BELGROW), which is administrated by BESPEED since 1985. The Registry stores coded data and informed consent was secured prior to data entry. Data of patients who started GH treatment between January 2003 and May 2010 were analyzed.

Diagnosis of SGA or GHD was made by the treating physician after peer-review by the other BESPEED members. All GHD patients had a peak GH concentration ≤ 10 μg/L in two provocation tests (glucagon and insulin test). Priming before testing with respectively estrogen and testosterone was done routinely in girls ≥8 years old and boys ≥9 years old. GHD patients with and without developmental anatomical anomalies of the pituitary were included. Patients with acquired GHD were excluded. Severe GHD was defined as a peak GH response less than 5 μg/L in both provocation tests. Included SGA children had a birth weight and/or birth length < − 2 SD [23] and a height < − 2.5 SD at the age of 4 years and at onset of therapy. Prepuberty was defined as having a testicular volume less than 4 ml for boys and Tanner breast stage 1 for girls.

In the GHD group, patients born SGA were excluded. In the SGA group, patients with severe GHD (peak GH < 5 μg/L) were also excluded. Additional exclusion criteria for all groups were: age ≥10 years for girls and ≥12 years for boys at the end of the first year of GH treatment, gestational age <30 weeks, any chronic disease or genetic syndrome interfering with a normal growth potential, a known poor adherence to GH treatment, concomitant treatment with steroids > 12 mg/m^2^.day (hydrocortisone equivalent), additional previous or current growth promoting therapy such as sex steroids, oxandrolone or aromatase inhibitors. Only patients who remained prepubertal during the first treatment year were considered for analysis.

### Methods

Variables retrieved from the register were (a) status at birth: gender, birth weight and length; (b) genetic background: mother’s height (Ht), father’s Ht; (c) patient variables at the start of the treatment period: chronological age, Ht, weight (Wt), the highest peak GH concentration in GH provocation tests; (d) first year GH treatment modality: average GH dose (μg/kg.day) during the first year of GH treatment; (e) Ht, Wt after 1 year of GH treatment. IGF-1 values (ng/mL) before the start and during the first year GH treatment were retrieved from the medical files.

Birth weight for gestational age was transformed into SDS, based on the standards of Niklasson et al. [23]. The midparental height (MPH) was calculated as follows: [father’s Ht (cm) + mother’s Ht (cm) + 13 for boys/− 13 for girls]/2 [24]. Height, weight, body mass index (BMI), HV, and MPH were converted to SDS using Belgian reference data by Roelants et al. [25].

First-year gain in height (∆Ht) SDS and first-year height velocity (HV) (cm/year), were calculated as the increment in height between start of treatment and a measurement made after minimum 9 months and maximum 15 months of GH treatment, subsequently scaled to 12 months.

The observed first-year HV (cm/yr) was expressed as SDS, either using the Flemish HV reference curve [25] or using the reference curves for GH treated prepubertal GHD and SGA children developed by Ranke et al. based on the KIGS database [13]. The latter was calculated as follows: the HV of the child in cm/year minus the mean HV for age and diagnosis divided by the SD for age and diagnosis.

Predicted first-year HV (cm/year) was calculated with the KIGS first-year prediction models for GHD (GH peak included) [18, 19] and SGA [20], provided that all parameters required for the mathematical algorithm were available. These prediction models are available on the Prediction Models Web [26] and on the iGRO website [27]. Differences between observed and predicted HVs were expressed as studentized residuals (SR). SRs were calculated as the observed HV minus the predicted HV, divided by the SD of the predicted HV of the child. SR is the index of responsiveness (IoR), thus the index of an individual’s actual growth versus its unique predicted growth.

The criteria used to define a poor first-year growth response were: (a) ∆ Ht < 0.3 SD [28], (b) ∆ Ht < 0.5 SD [13], (c) HV < + 0.5 SD on the population HV reference curves [25], (d) HV < + 1.0 SD on the population HV reference curves [25], (e) observed first-year HV more than 1 SD below the patient’s predicted first-year height velocity (SR < − 1) [12], and (f) HV < − 1.0 SD for expected first-year height velocity based on diagnosis specific reference data developed by Ranke et al. (HV Ranke SDS) [13].

The patients were divided into three response groups: poor response to all criteria, questionable response (poor response to at least 1 criterion), and good response to all criteria.

The percentage increase in IGF-1 was calculated using the IGF-1 value before the start of GH treatment and the highest IGF-1 value during the first year of GH treatment. A poor IGF-1 response during the first year of GH treatment was defined as an increase of less than 40% after at least 3 months of GH therapy. This cutoff value corresponds to the 10th percentile of IGF-1 increase in GHD patients [29].

### Statistical analysis

The variables were tested for normality with the one-sample Kolmogorov-Smirnov test and are reported as medians (25–75 percentile) or means (± SD). Student’s t test, one-way ANOVA and Bonferroni correction were used to test for differences between groups when the distribution of data was normal. Otherwise Mann-Whitney-U and Kruskal-Wallis tests were applied. Simple linear correlation analysis was conducted using the Spearman formula. Statistical significance was set at the 5% level (*p* < 0.05). IBM SPSS statistics 21® software was used for all statistical analyses. The patient population was of sufficient size to detect a 50% lower percentage poor responders for the criterion SR < − 1 compared to the criterion HV < + 1.0 SD (α = 0.05 and 1-β = 0.8).

## Results

### Baseline characteristics

In total, 171 SGA patients and 122 GHD patients met the inclusion and exclusion criteria. Sixty six children were diagnosed with severe GHD (peak GH < 5 μg/L). Baseline auxological characteristics at the start of GH treatment are listed in Table 1. For both groups there was a predominance of males (64–66%). Boys started GH treatment at a significantly older age than girls (7.5 vs. 6.6 years, respectively; *p* = 0.01); 26 (=15%) SGA and 19 (=16%) GHD boys were older than 10 years at the start of treatment. At baseline, there was no significant difference in median Ht SDS between SGA and GHD patients (SGA: − 3.06 SD, GHD: − 3.20 SD). The mean average GH dose during the first year of GH treatment for patients with GHD was 26.9 μg/kg*day, which was significantly lower than the dose for patients with SGA (38.1 μg/kg*day; *p* < 0.001). Children born SGA had a lower weight and BMI at start than children with GHD (*p* < 0.001). Children with GHD had the largest difference between height SDS at start and MPH SDS.

**Table 1:** Characteristics: background, at GH start, after first-year GH treatment

	SGA						GHD (all)						Severe GHD (peak GH < 5 μg/L)						Less-severe GHD (peak GH 5–10 μg/L)					
	*n*	Mean	SD	Median	p25	p75	*n*	Mean	SD	Median	p25	p75	*n*	Mean	SD	Median	p25	p75	*n*	Mean	SD	Median	p25	p75
Background																								
Birth weight, SDS	169	−2.56^1^	0.96	−2.50	− 2.93	− 2.03	121	− 0.44^1^	0.9	− 0.43	− 1.21	0.27	65	− 0.19	0.94	− 0.14	− 0.85	0.47	56	−0.74	0.78	−0.77	−1.35	− 0.13
Birth length, SDS	163	−2.77^1^	1.02	− 2.65	−3.41	− 2.19	112	−0.59^1^	0.89	−0.58	−1.23	−0.03	61	−0.48	0.87	−0.55	−1.1	0.17	51	−0.71	0.91	− 0.83	−1.4	−0.28
Sex, % male	64						66						64											
Father height, SDS	164	−1.40^2^	1.12	− 1.48	−2.1	−0.47	118	−0.85^2^	1.27	−0.97	−1.66	− 0.15	64	− 0.72	1.32	−0.67	− 1.65	0.24	54	−0.99	1.2	−1.05	− 1.95	− 0.26
Mother height, SDS	163	−1.37^1^	1.11	−1.28	−2.12	−0.61	119	−0.77^1^	1.13	−0.78	−1.53	0.07	64	−0.82	1.16	−0.78	−1.55	0.07	55	−0.72	1.11	−0.61	−1.45	0.07
MPH, SDS	163	−1.39^1^	0.85	−1.42	−1.95	−0.85	118	−0.82^1^	0.92	−0.82	−1.56	−0.27	64	−0.96	1.29	−0.96	−1.99	0.02	54	−1.05	0.98	−0.83	−1.87	−0.4
Maximum GH peak, μg/L							122	4.9	2.6	4.8	3.0	7.1	66	2.9	1.4	3.0	1.9	4.1	56	7.4	1.4	7.3	6.0	8.4
At start GH treatment																								
Age, years	171	7.5	2.3	7.5	5.4	9.2	122	6.7	2.9	6.5	4.4	8.8	66	6.5	3.1	5.9	4.2	8.6	56	6.96	2.8	7.7	5.3	8.9
Height, SDS	171	−3.21	0.65	−3.06	−3.51	−2.77	122	−3.17	0.83	−3.2	−3.69	−2.71	66	−3.33	0.89	−3.32	−3.8	−2.81	56	−2.98	0.7	−2.9	− 3.43	− 2.59
Weight, SDS	168	−3.34^1^	1.21	−3.29	−4.1	−2.47	120	−2.53^1^	1.19	−2.44	−3.36	−1.69	65	−2.57	1.22	−2.49	−3.34	−1.77	55	−2.48	1.15	−2.4	−3.4	− 1.6
BMI, SDS	167	−1.28^1^	1.2	−2.21	−2.13	−0.41	113	−0.33^1^	1.13	−0.52	−1.06	0.4	62	−1.17	1.04	−0.46	−0.85	0.63	51	−0.53	1.22	−0.74	−1.18	0.27
Height SDS minus MPH SDS	163	−1.83^1^	0.87	−1.75	−2.28	−1.21	118	−2.36^1^	1.14	−2.26	−3.04	−1.51	64	−2.58	1.27	−2.41	−3.31	−1.7	54	−2.11	0.9	−2.12	−2.66	−1.42
GH dose, μg/kg*day	167	38.1	7.2	36.5^1^	33.5	41.2	120	26.2	3,0	25.8^1^	24.4	27.4	65	25.8	3.1	2.3	23.8	27.3	55	26.6	2.9	26,0	24.7	27.4
After first-year GH treatment																								
Height, SDS	171	−2.64	0.71	−2.48^2^	−2.98	−2.15	122	−2.35	0.75	−2.32^2^	−2.75	−1.91	66	−2.35	0.78	−2.31	− 2.92	−1.77	56	− 2.35	0.71	− 2.34	−2.69	− 2.05
∆ BMI, SDS^a^	167	0.18^1^	0.47	0.15	−0.15	0.44	120	− 0.21^1^	0.59	− 0.19	− 0.54	0.18	65	− 0.27	0.68	− 0.23	− 0.57	0.2	55	− 0.14	0.47	− 0.14	− 0.48	0.17
Height SDS minus MPH SDS	163	−1.25^1^	0.91	− 1.15	− 1.78	−0.59	118	− 1.52^1^	0.89	− 1.53	−2.19	− 0.89	64	− 1.58	1.01	−1.61	−2.47	− 0.89	54	− 1.45	0.72	− 1.5	− 1.97	− 0.94
GH dose during first year, μg/kg*day	167	38.1	6.7	36.4^1^	34,0	41.6	119	26.9	3.2	26.5^1^	25,0	28.2	64	26.6	2.9	26.4	24.9	28.2	55	27.3	3.5	26.5	25,0	28.4

*SGA* small for gestational age, *GHD* growth hormone deficiency, *GH* growth hormone, *MPH* midparental height, *BMI* body mass index; ^a^ Increase in BMI during first-year GH treatment; ^1^*p* < 0,001; ^2^*p* < 0,05

### Response and responsiveness after the first year of GH treatment

As shown in Table 2, children with GHD had a significantly greater increase in Ht SDS and HV than children with SGA (*p* < 0.001). In the SGA group the mean observed HV is close to the expected HV. In contrast, GHD responded slightly worse than predicted (SR − 0.35 ± 1.13; *p* = 0.05).

**Table 2:** First-year response and responsiveness to GH treatment

	SGA						GHD (all)						Severe GHD (peak GH < 5 μg/L)						Less-severe GHD (peak GH 5–10 μg/L)					
	*n*	Mean	SD	Median	p25	p75	*n*	Mean	SD	Median	p25	p75	*n*	Mean	SD	Median	p25	p75	*n*	Mean	SD	Median	p25	p75
Response parameters																								
∆ Height, SDS^a^	171	0.57	0.26	0.56^1^	0.38	0.77	122	0.83	0.59	0.69^1^	0.47	1.08	66	0.97	0.65	0.88	0.55	1.31	56	0.62	0.41	0.59	0.38	0.74
HV, cm/year	171	8.1	1.5	8.2^1^	7.2	9.2	122	9.4	2.6	9.0^1^	7.6	10.9	66	10.1	2.8	9.9	8.4	12,0	56	8.4	1.9	8.3	7.2	9.5
HV for age and sex, SDS	170	1.98	1.42	2.04^2^	1.01	2.91	105	2.7	2.03	2.52^2^	1.27	3.72	57	3.22	2.33	3.06	1.41	4.9	48	2.09	1.39	2.1	0.97	2.78
Parameters of estimated responsiveness																								
HV Ranke^b^, SDS	171	−0.09	0.85	−0.08	−0.67	0.55	118	0,00	0.87	0.04	−0.57	0.53	63	0.03	0.92	−0.02	−0.47	0.64	55	−0.04	0.81	0.04	−0.58	0.37
Studentized residual	159	−0.12	0.94	−0.02	−0.7	0.5	113	−0.35	1.13	0.26^3^	−0.89	0.2	60	−0.43	1.3	−0.4	−0.93	0.3	53	−0.25	0.9	−0.16	−0.89	0.17

*SGA* small for gestational age, *GHD* growth hormone deficiency, *GH* growth hormone, *HV* height velocity; ^a^gain in height SDS after first-year GH treatment; ^b^KIGS growth targets for first-year GH response; ^1^*p* < 0,001; ^2^*p* < 0,05; ^3^*p* < 0,05 vs. zero

Children with severe GHD (max. GH peak < 5 μg/L) had a greater increase in Ht SDS (0.97 ± 0.65 vs 0.62 ± 0.42; *p* = 0.001), and a greater HV (cm/yr) (10.1 ± 2.8 vs. 8.4 ± 1.9; *p* < 0.001) than the group with less-severe GHD.

There was no significant difference in ∆Ht SDS, nor in HV (cm/yr) between SGA children with only a low birth weight (*n* = 24) and SGA children with only a low birth length (*n* = 38).

### Comparison of poor response and poor responsiveness criteria

**Fig. 1:**
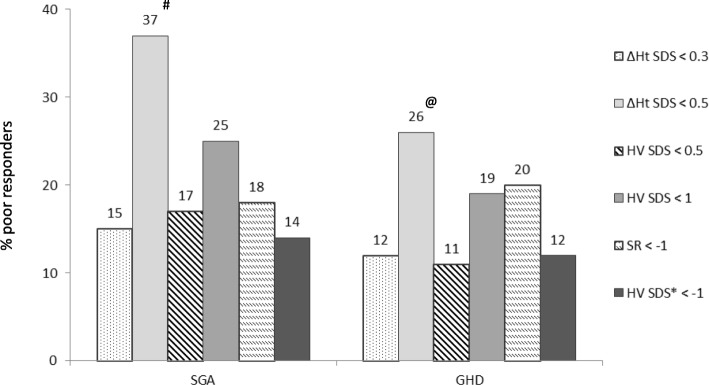
Percentage of poor growth responders after first-year GH treatment in prepubertal children according to various criteria in SGA and GHD patients. SGA = small for gestational age; GHD = growth hormone deficiency; SDS = standard deviation score; ∆Ht = first-year gain in height; HV = height velocity; SR = studentized residual. * HV < − 1 SD for expected first-year treatment response based on reference data developed by Ranke et al. ^#^ *p* < 0,01 vs. ∆Ht SDS < 0.3, HV SDS < 0.5, SR < − 1, HV Ranke SDS < − 1; ^@^ *p* < 0,05 vs. ∆Ht SDS < 0.3, HV SDS < 0.5, HV Ranke SDS < − 1

One hundred and six (106) patients (=36%) met at least one of the proposed criteria for poor response. Figure 1 shows the percentage of patients labeled as poor responders according to the different criteria. ∆Ht < 0.5 SD gave the highest proportion of poor responders (37% in SGA, 26% in GHD). ∆Ht < 0.3 SD generated 15% poor responders in SGA and 12% in GHD. HV < 0.5 SD was seen in 17% of SGA and in 11% of GHD patients. HV < 1.0 SD was observed in 25% of SGA and 19% of GHD subjects. Eighteen percent of patients with SGA and 20% of patients with GHD had an observed first-year HV more than 1 SD below the predicted first-year height velocity (SR < − 1). Fourteen percent of patients with SGA and 12% of patients with GHD had a HV < − 1.0 SD for expected first-year HV based on diagnosis specific reference data developed by Ranke et al. (HV Ranke SDS < − 1).

Between the SGA and GHD group there were no significant differences in percentages of poor responders. In the SGA group, the percentage of poor responders for the ∆Ht < 0.5 SD criterion was significantly different from those for the ∆Ht < 0.3 SD, HV < + 0.5 SD, SR < − 1 and HV Ranke <− 1 SD criteria (*p* < 0.01). In the GHD group, the percentage of poor responders for the ∆Ht < 0.5 SD criterion was significantly different from those for the ∆Ht < 0.3 SD, HV < + 0.5 SD and HV Ranke <− 1 SD criteria (*p* < 0.05).

**Fig. 2:**
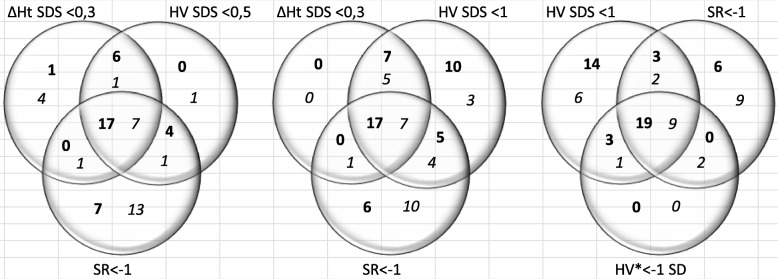
Number of poor responders for SGA patients (bold) and GHD patients (italic). SGA = small for gestational age; GHD = growth hormone deficiency; SDS = standard deviation score; ∆Ht = first-year gain in height; HV = height velocity; SR = studentized residual. * HV < − 1 SD for expected first-year treatment response based on reference data developed by Ranke et al.

Although the percentages of poor responders were comparable for the criteria ∆Ht < 0.3 SD, HV < + 0.5 SD, HV < + 1 SD, SR < − 1, and HV Ranke <− 1 SD in the SGA and GHD group, these specific criteria did not always identify the same patients as poor responders, as shown in Fig. 2. For example, for the criteria ∆Ht < 0.3 SD, HV < + 1 SD and SR < − 1, only 17/45 SGA patients and 7/30 GHD patients were identified as poor responders by all three criteria. For the criteria HV < + 1 SD and SR < − 1, respectively 22/45 and 11/30 patients in the SGA and GHD group were identified by both criteria as poor responders.

Poor response to all criteria was observed in 16 (10%) SGA and 7 (7%) GHD patients, questionable response (poor response to at least one criterion) in 49 (30%) SGA and 34 (32%) GHD patients, and good response to all criteria in 96 (60%) SGA and 64 (61%) GHD patients. In the SGA group, age was significantly older in the group with questionable response compared to the group with good response (data not shown). There were no other significant differences between the responder groups in the SGA group. In the GHD group, father height SDS was significantly lower and ∆ BMI SDS was significantly higher in the group with questionable response compared to the group with good response. There were no other significant differences between the responder groups. IGF-1 could not be compared because this parameter was available in only a minority of the patients (data not shown).

### IGF-1 response of poor growth responders

Out of the 106 patients who showed a poor growth response for at least one criterion, 70 patients had results of at least two IGF-1 determinations available. There were no significant differences in the growth responses between poor responders with and without available IGF-1 values. For SGA (*n* = 41) and GHD (*n* = 29) patients with a poor first year growth response, the mean increase in IGF-1 was 126% (±126) and 176% (±193), respectively.

Ten (24%) SGA and 4 (14%) GHD patients had less than 40% increase in IGF-1 during the first year of GH treatment. GHD patients with blunted IGF-1 increase had a significantly lower BMI SDS at start compared to those with a normal increase (− 1.35 SD vs − 0.21 SD; *p* < 0.01) and had mothers with a taller height (0.66 SD vs − 1.02 SD; *p* < 0.01), while no differences in the available auxological parameters were found between SGA children with a poor and a normal IGF-1 increase.

## Discussion

Depending on the criteria used, between 11 and 26% of short prepubertal GHD children, treated with a mean GH dose of 27 mcg/kg*d and between 14 and 37% of short prepubertal SGA children, treated with a mean GH dose of 38 mcg/kg*d, were found to be poor responders. ∆Ht > 0.5 SD was the most stringent criterion: 26% of GHD and 37% of SGA patients treated in Belgium did not meet this response criterion, whereas the HV Ranke SDS < − 1 gave the lowest percentages (12 and 14%).

Our prevalence results are comparable to the findings of Bang et al. [1] who also assessed the criteria for poor growth response in a group of 173 GHD and 54 SGA short prepubertal children from the Nordic countries. Beside the inclusion of SGA born children within the GHD group, the in- and exclusion criteria of this Nordic study are comparable to the data in our Belgian registry study, explaining to a great extend the similar proportion of poor responders.

Bang et al. [30] have argued that the response to GH should be clinically meaningful, implicating that treatment should diminish rapidly the height difference with peers, implicating a gain in height SDS of at least 0.5 SD during the first year. This criterion is based on the observation that the year to year change in height SDS in normal growing children can go up to 0.3 SD [31]. So to attribute the growth response to GH, the change in height SDS should be at least higher than 0.3 SD. However, since the gain in height SDS is age and diagnosis dependent [1, 16], a fixed cutoff will favor a better response in younger children and in severe GHD.

Comparing the annualized HV during the first year on GH with the HV of the pre-treatment year (∆HV, cm/year) might give an approximation of the GH induced HV, except in case a severely HV declining in the pretreatment year is present, as often seen in severe GHD. Theoretically, ∆HV (cm/year) may be the best response parameter to evaluate, however reliable pretreatment height measurements are often unavailable, as was the case in our database.

HV (cm/year) during the first year on GH treatment is highly age dependent [1, 16]. To express HV independent of age and in relation to normal gender related reference values, an SDS for age can be calculated. However, references are usually based on longitudinal studies with relatively small sample sizes or on cross-sectional data.

The ability of an individual patient to respond to GH (the responsiveness) should always be determined in order to evaluate the growth response correctly. For example, a patient with a first-year ∆Ht of 0.7 SD would be considered a good responder, but with a SR of, for example, − 1.2 this patient proves to have an inadequate response. A weakness of prediction models may be the lack of available patient characteristics needed to calculate responsiveness.

We hypothesized that a more individualized responsiveness criterion would yield 50% less poor responders than the more general response criteria. This hypothesis must be rejected because ∆Ht < 0.3 SD, HV < + 0.5 SD, HV < + 1 SD, SR < − 1 and HV Ranke SDS < − 1 SD gave the same proportion of poor responders in both treatment indications, although the GH doses are significantly different in both diagnostic groups. This supports the notion that there exists a continuum and overlap between partial GHD and SGA children without a postnatal catch up growth [32].

Although most criteria resulted in the same proportion of poor responders they did not identify the same patients. For example, HV < + 1 SD, the reimbursement response criterion of the European Medicines Agency (EMA) for GH treatment in short SGA children, generated a comparable amount of poor responders as the criterion SR < − 1 (respectively 25 and 18%). However, only 17 out of 45 of these poor responders fulfilled both criteria. Hence, these parameters cannot be used interchangeably. The fact that there is no concordance between the groups defined by the different criteria is interesting, but not surprising, since the response variables are principally different from the responsiveness parameters .

The long-term evaluation of response to GH has been validated for the KIGS prediction models by showing that SR is the second most important predictor of adult height after GH treatment. All the other proposed criteria for a poor first-year response have not been evaluated for their ability to predict a poor adult height outcome.

In our study, respectively 24 and 14% of the poor responders in the SGA and GHD group were found to have an insufficient IGF-1 increase in the first year. GH insensitivity is hence not a major reason for poor growth response in these children. GHD patients with low IGF-1 increase had a significantly lower BMI SDS at start compared to those with a normal increase. Nutritional constraints are possibly an important cause for the poor IGF-1 response. These children do not have sufficient calories to be able to grow, which may explain the poor growth response. Poor compliance is another possible reason for the poor IGF-1 response and growth response. Because IGF-I rises within days after GH administration, a normal IGF-I measurement cannot rule out poor adherence up to a week before the blood collection.

A weakness of this study is the limited amount of available IGF-1 values. However, no significant differences in the growth responses between poor responders with and without available IGF-1 values were observed.

IGF-1 levels after GH might fluctuate with the duration of GH therapy [1]. We therefore have chosen to take the maximum level into account and not a level at fixed duration. To circumvent the problem of non-centralized determination of IGF-1, the percentage increase was calculated on IGF-I levels determined in the same laboratory.

## Conclusions

In conclusion, with the exception of the ∆Ht < 0.5 SD cutoff, the tested criteria resulted in the same proportion of poor growth responders in GH treated SGA and GHD patients, but did not always identify the same patients as poor responders. This study does not provide evidence that one criterion is better than another. A critical evaluation of these response parameters and their cutoff values with respect to their capacity to detect a poor final adult height outcome is needed to define the best poor response parameter. A limited capacity in IGF-1 generation did not appear to be a major reason for a poor growth response in both GHD as SGA children.

## Abbreviations

∆Ht: Change in height
∆HV: Change in height velocity
BESPEED: BElgian Society for PEdiatric Endocrinology and Diabetology
BMI: Body mass index
EMA: European Medicines Agency
GH: Growth hormone
GHD: Growth hormone deficiency
Ht: Height
HV: Height velocity
IoR: Index of responsiveness
MPH: Midparental height
SDS: Standard deviation score
SGA: Small for gestational age
SR: Studentized residual
Wt: Weight
